# Approaches to Enhance the Potency of Vaccines in Chickens

**DOI:** 10.3390/vaccines12121337

**Published:** 2024-11-27

**Authors:** Oenone Bodman-Harris, Christine S. Rollier, Munir Iqbal

**Affiliations:** 1 Avian Influenza and Newcastle Disease Research Group, The Pirbright Institute, Ash Road, Woking GU 24 0NF, UK; oenone.bodman-harris@pirbright.ac.uk; 2 School of Biosciences, Faculty of Health and Medical Sciences, University of Surrey, Guilford GU2 7XH, UK; c.rollier@surrey.ac.uk

**Keywords:** poultry, vaccine potency, antigen-targeted vaccine, nanotechnology, probiotics, adjuvants

## Abstract

Outbreaks of avian pathogens such as Newcastle disease virus, avian influenza virus, and salmonella have a major impact on economies and food security worldwide. Some pathogens also pose a significant zoonotic potential, especially avian influenza viruses. Vaccination plays a key role in controlling many poultry diseases, and there are many vaccines licenced in the United Kingdom for diseases of poultry caused by viruses, bacteria, and parasites. However, these vaccines often do not provide complete protection and can cause unwanted side effects. Several factors affect the potency of poultry vaccines, including the type of vaccination used, the mechanism of delivery, and the use of adjuvants. Advancements in technology have led to the study and development of novel vaccines and vaccine adjuvants for use in poultry. These induce stronger immune responses compared with current vaccine technology and have the potential to protect against multiple poultry diseases. This review aims to discuss the existing poultry vaccine technology; the effect of delivery mechanisms on vaccine efficacy; the use of current and novel adjuvants; the ability to target antigens to antigen-presenting cells; and the use of probiotics, multivalent vaccines, and nanotechnology to enhance the potency of poultry vaccines.

## 1. Introduction

Poultry meat is an important food source for many people, with over 16 million tonnes estimated to be traded in 2022. Poultry meat is also projected to account for over half of all additional meat produced in the next decade. However, the increase in the poultry meat trade is slowing down, with poultry meat production in 2022 rising by only 0.6% from 2021, the slowest ever pace on record [1,2]. The poultry industry also provides another valuable food source in the form of eggs, with egg production in 2020 exceeding 86 million metric tonnes. Global egg production is projected to increase 13% by 2029, with India and China accounting for nearly half of the global increase [2,3].

However, farmed poultry and associated egg production is at risk from severe diseases due to infection from viruses such as avian influenza virus (AIV), Newcastle disease virus (NDV), and Marek’s disease virus (MDV) and bacteria such as *Salmonella* [4,5,6,7]. Outbreaks of infections in poultry flocks is a leading cause of economic loss in the poultry industry worldwide as viral epidemics are often associated with reduced weight gain and decreased egg production, and they increase the risk potential for zoonosis events [3,8]. The decrease in poultry meat trade seen in 2022 is thought to be due in part to the severe widespread outbreaks of highly pathogenic avian influenza virus (HPAIV) that occurred in several of the major poultry-farming regions, including North and South America and Europe [2].

Vaccination is commonly used to control avian diseases and is primarily focused on the prevention of disease-associated morbidity and mortality rather than prevention of the infection itself [9]. Antimicrobial therapy against bacterial infections is also commonly used, both as a preventative measure and an active treatment in poultry farms. However, effective treatment requires a diagnosis of the specific infectious agent as well as knowledge regarding the dosage and potential interactions with other drugs. There is also a limited number of antimicrobial drugs that are commercially available for poultry; therefore, accurate diagnosis is key for effective disease treatment and a decrease in the associated risk of antimicrobial resistance development [10]. Together, this makes vaccination of poultry a safer and more economical method of preventing disease in poultry.

Preventative measures are being adopted to reduce disease outbreaks. These include diagnostics and surveillance; improvements in biosecurity; reduction in virus spread via the culling of infected and at-risk flocks; and better practices in housing, husbandry, vaccination, transport, slaughtering, and waste management [11]. However, poultry vaccines for some diseases, such as avian influenza, remain less effective at blocking virus transmission, allowing them to continue circulating in both vaccinated and unvaccinated flocks [8]. Therefore, there is a need to increase the potency of current and future vaccines in poultry.

Most vaccines used in poultry are attenuated or inactivated pathogens (Table 1). However, there are issues with using these technologies, as live attenuated vaccines can lead to reversion back to more virulent strains, thus negating the vaccination in the first place. Live attenuated vaccines are also associated with adverse reactions, especially when vaccinating young chicks [12]. Another important issue to consider is the need to distinguish between infected and vaccinated animals (DIVA), which is not possible when using attenuated or inactivated vaccines. In addition, attenuated or inactivated vaccines can sometimes become ineffective due to the presence of maternally derived antibodies (MDAs) in young chicks, and so, vaccination may have to be delayed until they are older [12,13].

## 2. Current Poultry Vaccine Technology

### 2.1. Current Use of Inactivated Virus Vaccines in Poultry

One of the oldest methods of vaccine preparation is that in which the native seed virus is cultured in embryonated chicken eggs or in cell culture. Cultured viral particles are then inactivated chemically (e.g., with formalin, binary ethylenimine, or β-propiolactone) or physically (e.g., heat or gamma or ultraviolet radiation) to destroy infectivity whilst preserving immunogenicity. This method is still used to make up most registered avian influenza vaccines for poultry (Table 1) [3,14]. Inactivated vaccines are safer than live vaccines; however, they often induce low immunogenicity, thus requiring booster doses and formulation with adjuvants (e.g., oil emulsion) for long-lasting immunity [15,16]. The persistence of immunity also is often impaired by the age of the bird when the vaccination is given. For example, when given immediately after hatching, the response is often lower than in birds immunised at 4 weeks old [17]. The immune response also has a slow onset and is not DIVA compliant and, in young animals, protection can be decreased due to interference from the MDA [3].

### 2.2. Research and Current Use of Live Attenuated Vaccines in Poultry

Conventional attenuation is typically achieved via the serial passaging of the wildtype pathogen in either an irrelevant cell culture or host and has a long history of use in vaccine production against several poultry diseases (Table 1). Live attenuated vaccines are cost effective due to minimal scale-up and processing costs and induce both humoral and cellular immunity. However, the attenuation process has a long turnaround time, and there is a risk of reversion to the initial wildtype form or recombination with circulating pathogenic strains. More recently, reverse genetics has allowed for a more precise and faster method of generating live attenuated vaccines [3]. Coccidiosis is a parasitic infection of poultry caused by *Eimeria* spp. that is usually controlled by drug treatment; however, resistance is beginning to appear. To reduce the dependence on drug treatment, live attenuated vaccines have been developed via continuous passage in eggs. However, these vaccines lose effectiveness over time, resulting short expiry dates, and they have the potential to revert back to the more virulent strain [18]. Such live attenuated vaccines have also been developed for protection against HPAIV in poultry. Attenuation was achieved via the removal of the haemagglutinin (HA) polybasic cleavage site. Further attenuation was also carried out via genetic modification to truncate the NS1 genes. The study showed that vaccines carrying truncated versions of HPAIV NS1 genes completely protected poultry from homologous and heterologous virus challenges [19].

### 2.3. Research into Using Subunit Vaccines in Poultry

It is not always necessary to include the whole pathogen in the vaccine formulation, as some antigens alone can induce protective immune responses. Recombinant subunit vaccines are DIVA compatible, stable, well established, and are considered safe as they do not contain live viral components, which, in turn, also reduces the likelihood of severe side effects [3,20]. Subunit vaccines can also be engineered to produce responses to several antigens either against serotypes of the same pathogen or against multiple pathogens. The use of several antigens has been shown to increase vaccine efficacy, as one antigen is not always sufficient at producing a protective immune response [21,22]. However, subunit protein vaccines also tend to have a low immunogenic response, as they can lack the pathogen-associated molecular patterns (PAMPs) that are present on the whole virus. To overcome this, subunit vaccines require high dosages, adjuvants, and frequent boosters to maintain the protective response [3,23]. The selected antigens can be presented in several types of novel vaccine platforms described below.

### 2.4. Research into Virus-like Particles (VLPs) Vaccines in Poultry

VLPs are non-infectious structures that are made up of viral structural proteins. VLP vaccines have been successfully used in humans against hepatitis B virus and human papillomavirus [24,25]. VLPs have also been shown to activate dendritic cells and so can stimulate both cellular and humoral immunity [26]. No VLP-based vaccines have been licenced for use in poultry. VLPs can be manufactured using eukaryotic and prokaryotic expression systems. VLP vaccines are considered a safer alternative to live inactivated and attenuated vaccines due to their lack of genetic material [27]. Experimental studies have shown high potency of protection in chickens against viral infections such as AIV and NDV [28,29]. A bivalent VLP containing the HA and matrix 1 (M1) proteins of HPAIV, H5N1, and the NDV fusion (F) protein was used to vaccinate chickens. The results showed that a single immunisation induced high levels of anti-NDV antibodies and haemagglutinin inhibition (HI) antibody titres against H5N1 AIV [30]. However, VLP production has a high cost associated with its expression, purification, and cold chain storage and reduced stability in field conditions, meaning their commercial use is so far limited [3,27].

### 2.5. Research into Using DNA Vaccines in Poultry

DNA vaccines are based on plasmids, which encode the vaccine antigen. The antigen is produced by the cells of the vaccinated animal. DNA vaccines can encode genes for the expression of specific or multiple antigens to provide protection against several different pathogens or serotypes of the same pathogen. Chickens were vaccinated with DNA plasmids encoding for the infectious bronchitis virus (IBV) proteins S1, N, or M, alone or in combination. The combination vaccine induced significantly higher antibodies compared with the monovalent vaccines [31]. DNA vaccines can also be designed to trigger specific cell-mediated immune responses [21]. However, the plasmids used to generate DNA vaccines may contain antibiotic resistance genes, which confers a risk of the transmission of antibiotic resistance to farmed birds [32]. Although the DNA vaccines were first studied in poultry in 1993 [33], only one poultry DNA vaccine has been granted a conditional licence in the USA, against H5 HPAIV [3,34]. There are several reasons why DNA vaccines are not reaching the commercial stages. DNA vaccines are often administered intramuscularly and require several doses to induce protection; this is not practical in field settings. In addition, studies tend to use SPF birds; therefore, in the field, protection may be reduced due to the presence of MDAs [21,35].

### 2.6. Research into Using mRNA Vaccines in Poultry

The first proof-of-principle mRNA vaccine was published over 20 years ago in the mouse model using the mRNA of influenza nucleoprotein (NP) [36]. This technology has been further developed in recent years, especially for use in humans in response to the SARS-CoV-2 pandemic [37]. mRNA vaccines are considered to have a high safety profile, as they are non-integrating and non-infectious. Additionally, mRNA is naturally degraded in the cell; however, the rate of degradation can be regulated via delivery methods. Modifications can also be made to make the mRNA more stable and less likely to be degraded, and thus, more likely to be translated [38,39,40]. mRNA vaccines can also be administered repeatedly and have the potential for rapid and scalable manufacturing for emerging diseases and/or variants [40].

mRNA is the transitional stage between the translation of protein-encoding DNA in the cell’s nucleus and protein production by ribosomes in the cell’s cytoplasm. There are two major types of RNA-based vaccines currently being studied, non-replicating mRNA and self-amplifying RNA (saRNA). Non-replicating mRNA vaccines encode the antigen of interest as well as the 5′ and 3′ untranslated regions (UTRs). saRNA vaccines encode the antigen of interest and the machinery required for viral replication; this allows for intracellular RNA amplification and increased levels of protein expression. For optimal transcription, the mRNA products need to contain an open reading frame encoding the antigen of interest, flanking UTRs, a 5′ cap and a poly-A tail. This results in an mRNA product that resembles the naturally occurring processed mRNA molecules found in eukaryotic cell cytoplasm [40]. Exogenous mRNA is immunostimulatory itself as it can be recognised by innate immune receptors such as TLRs 3, 7, and 8 [41]. This is, in theory, an advantageous feature of using mRNA in vaccine technology, as it may provide an adjuvant activity which can drive dendritic cell maturation and therefore elicit strong B- and T-cell responses [40].

Proof of concept in poultry has been carried out in vitro using a luciferase encoding saRNA in lipid nanoparticles. Treatment of chicken organoids and cells with the saRNA–luciferase construct resulted in bioluminescent signals detectable in in vitro transfected chicken tracheal and cecal cells, showing that saRNA can effectively enter and replicate in poultry cells [42]. An in vivo study has since been carried out in SPF chickens against AIV. This study showed that an mRNA–lipid nanoparticle vaccine was safe in both embryos and chicks. Furthermore, birds inoculated with the mRNA-based AIV vaccine produced significantly higher antigen-specific antibody titres, increased IFN-γ responses, and decreased viral load in organs when challenged [43]. A significant issue for the use of mRNA vaccine technology is the cost associated with vaccine production and storage. mRNA vaccines typically require cold chain storage, which is not practical in field settings.

### 2.7. Research and Current Use of Recombinant Viral Vectored Vaccines in Poultry

There are several recombinant viral vectors commonly used to produce poultry vaccines (Table 1). These are turkey herpesvirus (HVT), fowlpox virus (FPV), adenovirus, infectious laryngotracheitis virus (ILTV), and MDV. The most commonly used of these are FPV and HVT as these are phenotypically stable, rarely transmitted horizontally, and do not revert to virulence [3,44]. HVT-based vaccines appear to confer a higher efficacy as they are not as affected by MDAs compared with FPV-based vaccines [45,46]. Recombinant viral vectored vaccines can be administered in ovo or via subcutaneous injection at one day old [47,48] and can also be modified to contain more than one type of antigen, which broadens the protective efficacy [49].

There are several licenced recombinant viral vector vaccines against NDV (Table 1). Originally these were based on an FPV vector, but more recently, HVT vectors expressing the fusion or haemagglutinin–neuraminidase NDV proteins have been used. NDV itself can also be used as a virus vector with the advantages that it can be used to create bivalent vaccines. Like HVT and FPV, NDV is a natural pathogen of birds, ensuring the delivery of the antigen to the target tissues, eliciting both cellular and humoral immunity [12,50,51].

### 2.8. Research into the Use of Nanotechnology in Poultry Vaccines

Nanoparticles are defined as materials with a dimension of less than 100 nm [52]. Nanoparticles can be used as delivery systems for vaccines where the vaccine antigen can be incorporated on the surface or encapsulated within the surface of a nanoparticle. When encapsulated within the nanoparticle, this allows for antigens to be stabilised, preventing degradation by host cell proteases and allowing for increased uptake and processing by the immune system upon administration [53]. The main targets for vaccination of poultry using nanoparticle technology are NDV and AIV, *E. coli*, and *Salmonella* [54].

Nanoparticles have therefore been found to increase the potency of vaccines. An Ag@SiO_2_ nanoparticle-encapsulated DNA vaccine has been tested against NDV in poultry. This method of vaccination resulted in both cell-based and humoral immune responses. Increased levels of serum antibody, IL-2, and IFN-γ secretion compared with the standard DNA vaccine alone were observed when administered intranasally. This study showed that the Ag@SiO_2_ hollow nanoparticle is a safe and effective delivery method for NDV DNA vaccines and can also induce mucosal immunity [55].

Chitosan is a non-toxic biodegradable natural polysaccharide derived from crustacean and insect exoskeletons. Mice vaccinated with chitosan containing a vaccine elicited increased levels of antigen-specific antibodies, antigen-specific splenic CD4^+^ T cells, and strong delayed hypersensitivity responses [56]. In poultry, chitosan and calcium phosphate particles have both been used in the study of an NDV vaccine. Nanoparticle-based vaccines result in high antibody titres in both the mucosa and blood compared with standard inactivated NDV vaccines. When challenged with a lethal dose of NDV, the chitosan-based vaccine induced better protection than the calcium phosphate-based vaccine [57]. An oral chitosan nanoparticle vaccine was also shown to induce both cellular and humoral immunity towards *Salmonella* when delivered in feed and drinking water. This vaccine contained both the highly immunogenic *Salmonella* outer membrane proteins (OMPs) together with the flagellin (F) surface protein, which acts as a TLR5 antagonist in the intestines of birds. This resulted in the induction of both a cell-mediated and humoral response in vaccinated birds. Analysis of the immune response post-vaccination showed increased expression of other TLRs, Th1 and Th2 cytokines in chicken immune cells, increased IgA and IgY responses, and reduced pathogen load upon live bacterial challenge [58].

Altogether, these studies show that nanoparticles can support both cellular and humoral protective immune responses in poultry and may allow increased stability of the vaccine to allow for administration in food and drinking water. In addition, nanotechnology is a promising field for increasing the potency of poultry vaccines, as some nanoparticles can also provide an adjuvant effect (for further information, see Section 4. The Use of Adjuvants in Poultry Vaccines) and can be used as carriers for many other novel vaccine technologies such as VLPs and liposomes for the encapsulation of mRNA vaccine technology.

Nanobodies are antibodies derived from camelid species and are unique, as they contain only functional heavy chains and no light chains; they are also known as heavy-chain-only antibodies (HCAbs). The heavy chain of these antibodies consists of one variable domain, making them highly specific; this is known as the VHH domain or nanobody [59]. The VHH domain of an HCAb can be cloned and recombinantly expressed as a nanobody. Nanobodies are ~15K da, ten times smaller than a conventional IgG antibody, but retain the same binding affinity [59,60]. Nanobodies have been tested as a method of poultry vaccination. Necrotic enteritis is a devastating disease caused by the *Clostridium perfringens* bacterium. Nanobodies targeting this bacterium’s NetB toxin and collagen-binding adhesion molecule were given in feed to chickens. Chickens that received the nanobody containing feed had only a 7.5% mortality when challenged compared with a 23.75% mortality in untreated chickens [61]. Furthermore, nanobodies developed against NDV had the ability to inhibit viral replication in chicken embryo fibroblast cells (DF-1), but they have not been trialled in vivo [62]. This shows that nanobodies are safe and effective in poultry at protecting against disease. They also can be administered in food, making large-scale application possible.

### 2.9. Current Poultry Vaccine Technology Conclusions

In conclusion, there are several platforms available to be used in poultry vaccine technology. However, most commercially available vaccines use live-based vaccines, which are then attenuated or inactivated (Table 1). This is most likely due to the ability of live vaccines to induce robust immune responses and can often be used in combination, allowing for protection against several diseases or strains. The production of live attenuated and inactivated vaccines is also a well-established process commercially. The ability to switch to new technology is harder as the scale-up of manufacturing takes time and money. This may make poultry vaccines more expensive, which may impact their field use.

## 3. Delivery Mechanisms

**Figure 1:**
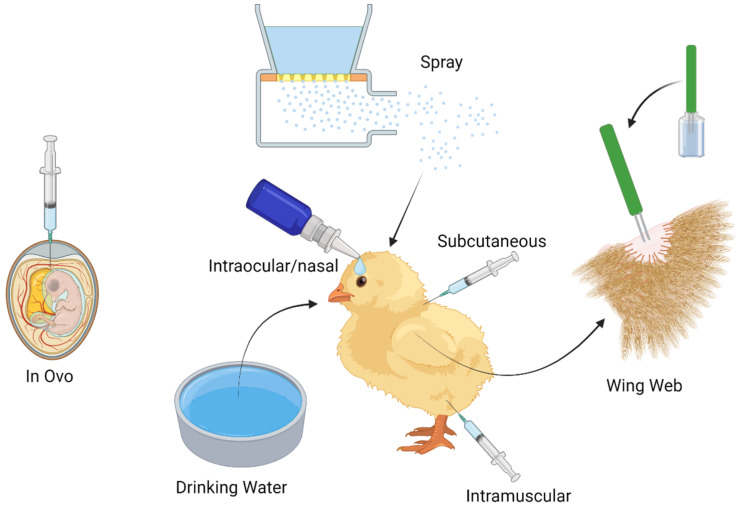
Methods used in the poultry industry for vaccinating chickens. Created with BioRender.com.

Effective poultry vaccination has become a challenge around the world due to the increase in demand for poultry meat and eggs, leading to more flocks and larger flock sizes. Vaccination has become more popular to carry out in the hatchery, where mass vaccination can be administered in ovo at embryo day 18–19, or by spray on the day of hatching. Vaccines can also be administered individually on the day of hatching via intramuscular or subcutaneous injection and intraocularly/nasally (Figure 1). Once at the farm, further vaccination can occur via spray, drinking water application, or wing web as well as intramuscular or subcutaneous injection (Figure 1). Although individual vaccination of animals is thought to be more effective, as all birds are vaccinated with the same known dose of antigen, it is becoming impractical due to the increasing number of commercial chickens worldwide.

### 3.1. The Current Use of in Ovo Vaccination in Poultry

In ovo vaccination can be carried out by puncturing a small hole through the blunt end of an egg. A needle is then used to deliver the vaccine to the amniotic cavity. This technique was developed in the 1980s [63]. Today, most of the commercial egg injection machines can vaccinate either the amniotic cavity or embryo body. Vaccination does not affect the hatchability or the performance of hatched chicks. These chicks have protective immunity from that hatch. In ovo vaccination is common in U.S. broiler hatcheries, and its popularity is expanding around the world [63]. Vaccines administered in ovo include MDV, IBD, HVT, ILT, NDV, and coccidiosis (Table 1) [63,64]. Although still in experimental stages, vaccines against AIV and *Mycoplasma gallisepticum* were found to be effective when administered in ovo [65,66].

The mechanism of protection elicited from in ovo vaccination is not fully understood, but both the innate and adaptive immune systems are thought to be involved. Nevertheless, there are several factors that influence the efficacy of in ovo vaccination. These include the site of injection, embryo development stage, and aseptic control measures in the hatchery, including the vaccination equipment and vaccine preparation [64]. In ovo vaccination occurs at embryonic incubation day 18, when all functional components of the immune system are developed in the chicken, allowing a sufficient immune response to be generated [63]. Vaccination of embryos via in ovo injection has been proven to be safe and effective with minimal adverse effects on hatchability. The site of injection is of particular importance; when MDV vaccine was administered to the allantoic sac, the vaccine was not effective, while delivery to the embryo body or amnion resulted in protection against MDV disease [67]. Determining the appropriate volume that is used in in ovo vaccination is also important, with suggested volumes not exceeding 2000 μL for electrolyte solution injection and 700 μL for carbohydrate solution injections. The commercially available MDV vaccine is administered in 50 μL of solution [64].

### 3.2. The Current and Future Use of Spray Vaccine Administration in Poultry

On the day of hatch, live attenuated IBV and NDV, avian metapneumovirus, and coccidiosis vaccines (Table 1) are administered as a coarse spray (droplets between 70 and 150 μM in hatcheries and between 100 and 150 μM in houses). Vaccines are usually diluted in distilled water to prevent any reactions with chlorine or natural salts in the water supply degrading the vaccine. Per application, spray equipment delivers 7–20 mL of diluted vaccine per 100 chicks [68]. Spray vaccination has lowered the costs associated with time and labour and is an efficient and effective method of vaccination. The size of the droplets means they land predominantly in the eyes and nostrils and are confined to the upper airway. The prevention of penetration into the lower respiratory tract reduces potential vaccine-associated adverse effects [9]. Although spray vaccinations are usually diluted in water, gel dilution and application has been studied and has been shown to be effective for coccidiosis vaccination [68]. Here, coccidiosis vaccine was gel diluted and then dropped onto chicks in the hatchery. There was an increase in oocyte shedding in the gel-administered group compared with the traditional water-diluted vaccine group. This suggests that the gel-administered birds received a higher vaccine dose. However, there was no difference in body weight of gross lesions between water-diluted or gel-diluted vaccinated birds when challenged [69].

Gel vaccination also reduces chicks becoming wet and therefore chilled during traditional spray vaccination. This, in turn, reduces the stress caused by vaccine administration [70].

Vaccines administered via coarse spray result in detectable immune responses from 3 days post-vaccination, with protective antibody titres present around 2–3 weeks post-vaccination [68].

### 3.3. Current Use of Drinking Water Vaccine Administration in Poultry

Vaccinating via drinking water also allows for application to many birds in a short space of time. It can be used to vaccinate against several poultry diseases including avian encephalomyelitis virus, chicken anaemia virus, coccidia, and *E. coli* (Table 1). To achieve successful vaccination, chemical and disinfectant residues need to be removed from the water line at least 2 days prior to vaccination, along with any other water treatment or medication [71]. To attain successful vaccination, birds are subjected to limited water availability two hours prior to the application of the vaccine to the drinking water to increase their thirst and, thus, likelihood of consuming the vaccine. In addition, the water containing the vaccine needs to be available for 2–4 h to ensure all birds have a chance to consume it [68].

### 3.4. Current Use of Subcutaneous/Intramuscular Vaccine Administration in Poultry

A subset of vaccines is recommended to be administered subcutaneously. This method was first introduced using live attenuated MDV vaccine and is still commonly used (Table 1) [9,68]. Inactivated viral and bacterial oil emulsion vaccines are commonly administered this way in several poultry species including turkey breeder pullets, broiler breeders, and layers before the onset of egg production (Table 1). In breeders, the use of inactivated vaccines is essential for the vertical transmission of antibodies to chicks for early protection, especially for protection against immunosuppressive viruses [72]. The individual handling of chickens for subcutaneous administration is, however, labour intensive, but it has proven effective for NDV and IBD vaccination [9].

### 3.5. Current Use of Intraocular/Nasal Drop Vaccine Administration in Poultry

Intraocular or intranasal vaccines are delivered using a manufacturer-provided dropper that delivers approximately 0.03 mL of vaccine solutions to the desired site (Figure 1). Dye can be added to the vaccine formulation to help determine which birds have been vaccinated in a given session. This method can present issues regarding cold chain storage, as the vaccine can be warmed by the hand of the vaccinator, decreasing vaccine efficacy. Although intraocular/nasal vaccination was popular in the early stages of large-scale poultry production, it has gradually been replaced by the use of drinking water and spray vaccination. However, vaccination against NDV, IBV, and avian rhinotracheitis can still be administered using this technique (Table 1) [68].

### 3.6. Current Use of Wing Web Vaccine Administration in Poultry

Wing web vaccination is commonly used in chickens and turkeys against FPV, avian encephalomyelitis (Table 1), chicken infectious anaemia virus, and *Pasteurella*. Here, the vaccine solution is applied to the wing web using an individual stabber with steel prongs containing small holes which hold the vaccine. Prongs must be submerged in the vaccine solution before each application (Figure 1). Seven days after vaccination, the formation of small nodule scabs that can be palpated at the site of inoculation are used to confirm successful vaccination; however, protection may vary depending on the breed of bird and vaccine applied [68].

### 3.7. Research into Intracloacal Administration of Poultry Vaccines

The bursa of Fabricius is the site of B-cell maturation and is unique to birds. The bursa of Fabricius is a diverticulum located near the cloaca and is the site where the antibody repertoire is developed [73]. Previously, the bursa of Fabricius was shown to be a channel through which environmental antigens can stimulate the immune system and induce a specific antibody response [74]. The uptake of particles by the cloaca (cloacal drinking) allows for the exposure of a wide variety of antigens to the lymphocytes present in the bursa of Fabricius [75]. Previously, it was shown that cloacal administration of a protein antigen did not produce a detectable serum antibody response in 4–6-week-old chickens. However, there was a generation of immunological memory for a subsequent challenge [76]. Intracloacal vaccination has been studied against IBDV, a virus which infects the bursa of Fabricius and results in immunosuppression after infection. Birds vaccinated intracloacally with a low dose of live attenuated IBDV resulted in increased IBDV antibody titres, and no immunosuppressive effect was seen against other vaccines given shortly after [77]. Intracloacal administration of live attenuated IBDV vaccine has also been shown to be able to overcome vaccine neutralization by MDAs [78]. Vaccines against *Clostridium perfringens*, a cause of necrotic enteritis, have also been investigated. Here, intracloacal administration was able to induce an increased inflammatory response in the cecal tonsils and other mucosal lymphoid organs [79]. However, it has also been shown that the administration of live and inactivated vaccines to 1-day-old chicks directly into the bursa of Fabricius via intracloacal administration did not generate a significant immune response [75].

Here it has been shown that intracloacal administration may be an effective method of protection against pathogens that primarily infect the intestinal mucosal tissues and associated organs. However, research into the age of bird of vaccination needs to be carried out as vaccinating too young may result in immune tolerance [75].

### 3.8. Delivery Mechanism Conclusions

Here we have shown that there are several different methods to vaccinate poultry, with each of them having their own positives and negatives. More research needs to be carried out on how different breeds of poultry respond to different vaccination methods, as birds that do not generate an immune response may act as reservoirs for some pathogens. There also needs to be more study into safe and effective mass application methods (e.g., spray, water/feed, and in ovo) due to the increasing flock size worldwide and the more general move towards automation in farming.

## 4. The Use of Adjuvants in Poultry Vaccines

Vaccine adjuvants are used to enhance the immune response to vaccines to allow for a lower dose per animal or to avoid the necessity of booster vaccinations. Adjuvants are often necessary to stimulate and direct both the innate and adaptive immune responses to otherwise poorly immunogenic vaccine antigens and can have more than one mechanism of action [13]. There are several adjuvants used in commercial and experimental poultry vaccines, which can be categorised into two groups: immunostimulant and delivery agents. Immunostimulants include Toll-like receptors (TLRs), saponins, and cytokines; these promote the secretion of proinflammatory cytokines, which simulate the antigen-presenting cells (APCs). Delivery agents, on the other hand, help preserve the antigen conformation for correct presentation to APCs and can also provide slow release for continuous stimulation of the immune system [80].

### 4.1. Current and Future Uses of Emulsions as Adjuvants (Delivery Agent and Immunostimulant)

**Figure 2:**
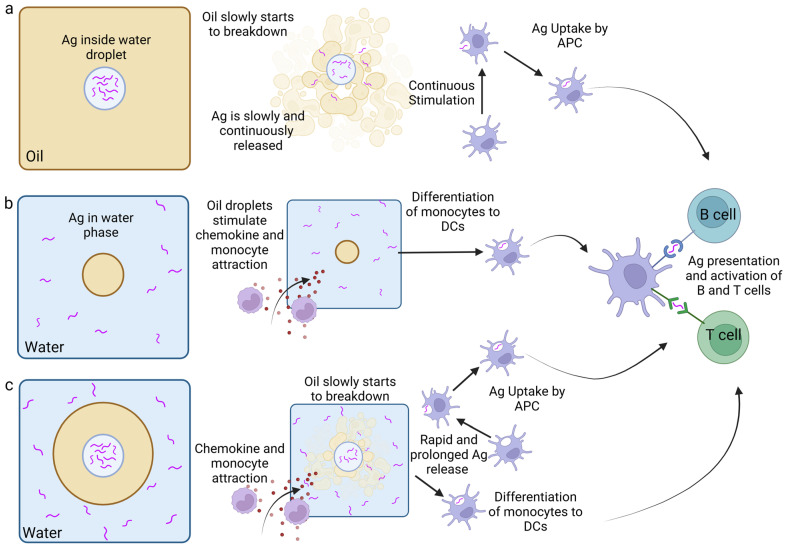
Illustration of different types of oil emulsions. (**a**) Water-in-oil emulsion (W/O). This method traps the antigen (Ag) inside water droplets. This protects the structure of the Ag, allowing for slow antigen release due to the breakdown of the oil. This prevents rapid clearance of the antigen, which allows for increased immune cell recruitment and antigen processing. (**b**) Oil-in-water (O/W) emulsion. Here, the oil helps attract monocytes and chemokines to the injection site. This promotes the differentiation of monocytes into dendritic cells (DCs) and stimulates a strong cellular immune response. (**c**) Water in oil in water (W/O/W). Here, the antigen is in both the oil and water phases, so the antigen is both taken up quickly from the water phase but also slowly released from the internal water phase. The breakdown of the oil also helps with the attraction of monocytes and chemokines to help induce a strong cellular immunity. Created with BioRender.com.

Emulsions have been utilised as adjuvant systems in animal vaccines for many years (Table 1) and are used frequently, as they show good efficacy in the production of antibodies and are cost-effective and easy to produce [80,81]. Using emulsions as an adjuvant allows the extended release of the antigen and enhanced antibody production, resulting in an overall increased immune response of the birds to the antigen [13]. Emulsions are formed from two immiscible liquids which, when combined, form small droplets of one liquid dispersed within the other. There are three types of emulsions used in vaccine formulations: water in oil (W/O), oil in water (O/W), and water in oil in water (W/O/W) (Figure 2) [80].

W/O emulsions involve the dispersion of water droplets in a continuous oil phase. Here, antigens are captured in the water phase and surrounded by a continuous oil phase. Therefore, the antigens are slowly released upon the breakdown of the oil after injection (Figure 2a). This preserves the antigen from fast clearance by the host, thereby increasing the time allowed for immune cell recruitment and antigen processing [81,82]. This is probably the most well-known type of emulsion adjuvant, and the prototype is known as Freund’s adjuvant, created in 1937. Freund’s adjuvant is based on a paraffin oil either with heat-killed and -dried mycobacteria (complete) or without mycobacteria (incomplete). It was found to be efficient at inducing a high antibody titre [80,83]. However, Freund’s adjuvant also resulted in high levels of adverse effects, so it is no longer used. More successful commercial W/O emulsions under the product name Montanide™ Incomplete SEPPIC Adjuvants (ISAs) (Seppic Korea Office, Seoul, Republic of Korea) have been developed, with lower adverse effects but comparable increases in efficacy, and are used in veterinary vaccines [80,84]. For example, W/O Montanide™ ISA has been successfully used as an adjuvant in vaccines against NDV and coccidiosis in chickens. The use of Montanide™ ISA as an adjuvant resulted in increased antibody titres against NDV compared with standard W/O emulsions as well as 100% protection when challenged [85]. Montanide™ ISA was used in the preparation of vaccines against *Eimeria acervuline*, one the causative agents of avian coccidiosis disease. When challenged, Montanide™ ISA adjuvanted vaccines resulted in increased weight gain and reduced parasite shedding compared with non-adjuvanted vaccines [86].

O/W emulsions are formed by the dispersion of oil droplets in the aqueous phase where the antigen is contained (Figure 2b). The oil droplets help chemokine-driven immune cell recruitment and differentiation of dendritic cells and macrophages. There are several commercially available O/W adjuvants developed by Montanide™ ISA and Emulsigen™. O/W adjuvants have been successfully used against equine and swine influenza, suggesting they can be used as adjuvants in vaccines against viruses in animals, but they have not been tested in poultry [80]. O/W emulsions have been used in human and veterinary vaccines. In humans, a subunit O/W MF59-adjuvanted vaccine against influenza H5N1 virus was shown to induce antibodies that are also cross-reactive in conjunction with antigen dose sparing [87,88]. In cattle, swine, and guineapigs, vaccines against foot-and-mouth disease were adjuvanted with commercially available O/W emulsions. These vaccines were more tolerant to long-term storage, and antibody levels were detectable for at least 6 months following vaccination [89].

In W/O/W emulsions, oil droplets containing internal water droplets are dispersed in a continuous water phase (Figure 2c). This has been suggested to provide a fast release of antigens from the external water phase coupled with a prolonged release due to the internal water droplets. Therefore, W/O/W may be able to facilitate both the quick and continuous stimulation of immune cells; however, few W/O/W adjuvants are available on the market. These are Montanide™ ISA 201 VG and 206 VG [80]. In poultry, vaccines against *Borrelia anserina*, the bacterium responsible for fowl spirochaetosis, have been studied using the commercially available Montanide™ ISA 206 VG emulsion. Chickens vaccinated with Montanide™ ISA 206 VG adjuvanted vaccines produced significantly higher antibody titres up to 42 days post-vaccination, whereas the non-adjuvanted vaccine saw a decrease in antibody titre from 28 days post-vaccination [90]. This suggests that W/O/W emulsions can be used in poultry vaccines to increase efficacy.

### 4.2. Research into Using Toll-like Receptors (TLRs) as Adjuvants (Immunostimulatory) in Poultry Vaccines

**Figure 3:**
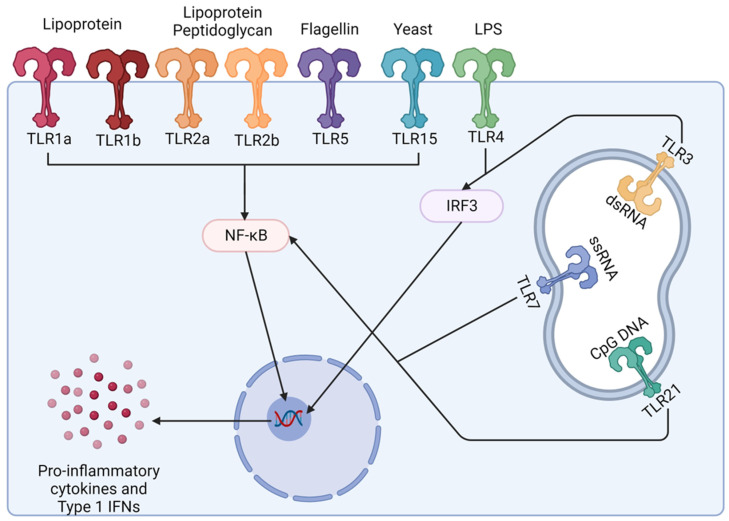
Overview of 10 known chicken TLRs. TLRs are anchored membrane receptor proteins that are expressed on APCs. They recognise conserved components on microbes and, once activated, result in a proinflammatory response and induce the expression of cytokines involved in immune cell differentiation. This helps the induction of cell-mediated and humoral immunity. IFN = interferon, TLR = Toll-like receptor, LPS = lipopolysaccharide. Created with BioRender.com.

Toll-like receptors (TLRs) are membrane-anchored receptors that are expressed in macrophages and dendritic cells and initiate host defence mechanisms upon recognising specific components of microbes. Once TLRs are activated, they have an important role in the proinflammatory response, and they contribute to the production of antigen-specific immunity by inducing the expression of cytokines involved in T-cell differentiation, and so, induce cell-mediated responses which, in turn, support the humoral immune response [80,91]. There are 10 known TLRs in chickens, and the use of TLRs as adjuvants in vaccines has shown promising results in several animal models, including poultry (Figure 3) [91,92].

Formalin-inactivated H5N2 vaccine adjuvanted with the TLR5 ligand *Salmonella* flagellin resulted in enhanced titres of the AIV-specific IgA antibody in vaccinated birds compared with the inactivated H5N2 vaccine alone. The increase in IgA antibodies makes this adjuvant of particular interest for use against pathogens that infect via mucosal tissues [93]. Ligands targeted by CpG oligodinucleotides (CpG-ODNs) (TLR21) and *Bacillus subtilis* spores were also shown to enhance host immunity against AIV with increased levels of specific IgA seen in the respiratory tract and serum IgG. Intranasal administration also resulted in an increased expression of proinflammatory cytokines in the trachea and nasal cavity [94]. Polyinosinic-polycytidylic acid (poly I:C) induces a TLR3 response because poly I:C acts as a dsRNA analogue that can induce a type I IFN response. Poly I:C has been added to chicken embryo cells before exposure to the NDV and avian reovirus [95]. Poly I:C treatment reduced plaque formation and induced an antiviral state against both viruses. Also, in NDV vaccines, CpG use increased serum IgG and IgA and T-cell proliferation when the NDV vaccines were administered intranasally, showing it can be used as an adjuvant to stimulate mucosal immunity [96]. The use of TLR ligand-adjuvanted vaccines has also been trialled with bacterial vaccines in poultry. Used in vaccines against *Salmonella enteritidis,* CpG-ODN, which targets TLR21, increased responses in chicken innate immune cells and resulted in a significant reduction in organ invasion by *Salmonella enteritidis* and subsequent associated mortality in challenge studies [97].

TLR adjuvants can reduce AIV shedding after prophylactic treatment with different TLRs; however, this response varied depending on the ligand and route of administration used [98]. An alternative method to deliver TLR ligands as vaccine adjuvants is by encoding the ligand sequences within a plasmid. CpG-encoded plasmids administered with the inactivated H5N2 vaccine in chickens induced significantly higher levels of IFN-y responses, and splenocytes expressed increased levels of TLR3 and TLR7 transcripts [99]. Encoding TLR adjuvants into plasmids offers a more cost-effective and large-scale production opportunity [100].

Therefore, TLRs can stimulate both a cell-mediated and humoral response, increasing the potency of vaccines in poultry in experimental trials. However, concerns have been raised about the induction of unwanted side effects, especially the induction of a strong inflammatory response when using TLRs as adjuvants in vaccines [91].

### 4.3. Research into Using Cytokines as Adjuvants (Immunostimulatory) in Poultry Vaccines

The induction of cytokines is a key mechanism of action for adjuvants; thus, the direct administration of cytokines could be used as an adjuvant to enhance vaccine responses. Additionally, cytokines could be used to directly bias host immune responses such as towards Th1 or Th2 cells. Methods for the administration of cytokines include encapsulation with liposomes, biodegradable polymers, conjugation with vaccine antigens, and cloning into genetic vaccine platforms. Several chicken cytokines have already been recognised to have antiviral and immunomodulatory properties [100].

Cytokines such as IFN-α, β, and γ as well as IL-1β have been studied as vaccine adjuvants in chickens. The combined administration of chicken IFN-α, IFN-γ, and IL-1β expressed in recombinant E. coli induced an increased antibody response against the tetanus toxin. However, there was no improvement in antibody response when recombinant chicken IFNs were co-administered with inactivated IBV vaccines [101].

Chicken INF-α has been widely studied as an immunomodulator and adjuvant in poultry vaccines [95]. Chicken INF-α produced in a prokaryotic expression system was evaluated in combination with the inactivated H9N2 AIV vaccine. This resulted in increased Th1 and antibody responses compared with the H9N2 vaccine alone [102]. Birds vaccinated with chicken IFN-α co-expressed with NDV in an FPV vector did not lose weight when challenged compared with birds that received the NDV vaccine alone. This also suggests that chicken INF-α treatment prevents disease due to its antiviral properties whilst still maintaining its vaccination potential, as protective responses were still observed up to 60 days post-vaccination when challenged [103].

IL-2 plays a significant role in the activation of T cells and NK cells. Adding recombinant IL-2 to an inactivated H5N2 AIV vaccine resulted in increased levels of IgG and IgA and increased levels of mast cells in the respiratory tracts of chicks when administered intranasally [104]. An H3N2 AIV that contained embedded IL-2 was used to generate an inactivated AIV vaccine wherein the virus particles also expressed surface IL-2. This vaccine induced increased systemic antibody levels compared with a conventional inactivated H3N2 AIV vaccine [105].

Plasmids expressing IL-15 have been used as genetic adjuvants in combination with an H5 AIV DNA-based vaccine. The H5 AIV DNA vaccine containing IL-15 plasmid resulted in increased blood CD8^+^ T cells and increased serum HI titres when administered at 14 days old compared with H5 AIV plasmid alone. Immune responses were seen in 1-day-old vaccinated chicks, though these were considerably lower. This shows that an IL-15 adjuvant increases both antibody- and cell-mediated responses, which may result in a potential increase in the protective effect against AIV infection [106]. Together, this shows that cytokines have potential as adjuvants in poultry vaccines.

### 4.4. Use of Adjuvants in Poultry Conclusions

In summary, there need to be larger-scale studies into the use of novel adjuvants in poultry. This is because the long-term health effects of these novel adjuvants are currently unknown. Therefore, longer and more in-depth bird health studies following vaccination with any novel adjuvant need to be carried out. Firstly, the adverse effects of all novel adjuvants need to be identified. This is especially important when regarding immunostimulatory adjuvants such as cytokines or TLRs. This is because these adjuvants are more likely to cause a systemic inflammatory response, which may affect the bird’s weight and/or egg production. If there is an effect on weight and/or egg production, this will make the new vaccines less likely to be licenced or used commercially due to the negative associated economic effects.

## 5. Targeting the Antigen to Antigen-Presenting Cells

Antigen-presenting cells (APCs) are a crucial part of the innate immune system and play an essential role in initiating the adaptive immune response. APCs such as dendritic cells (DCs) can induce the activation and clonal expansion of T cells. Therefore, targeting APCs via vaccination can help induce effective and long-term T-cell-based immune responses. The uptake of antigens by APCs using antigen targeting has been developed for use in humans to increase the potency of vaccines and immunotherapy treatments over the last two decades, especially to tackle cancer and autoimmune conditions [107].

**Figure 4:**
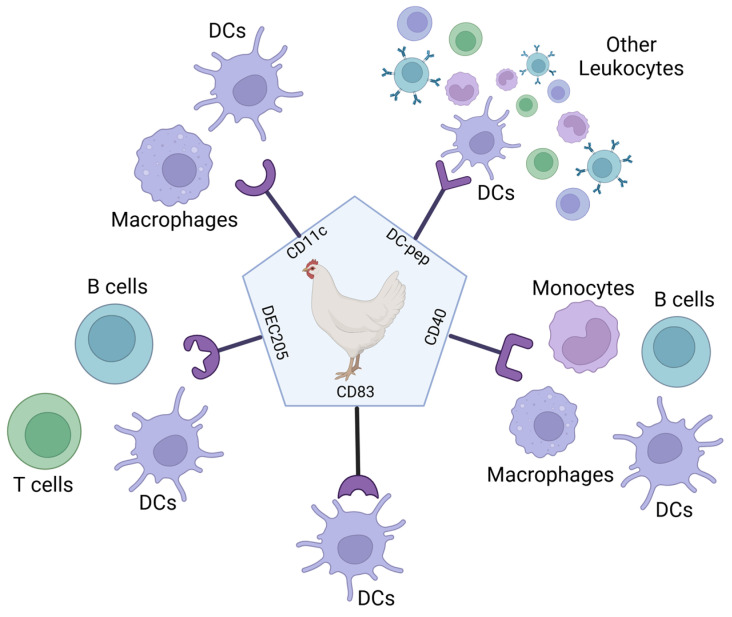
Antigen-presenting cell targets that have been validated in poultry vaccines. CD11c is a β-integrin molecule shown to be on mammalian dendritic cells, neutrophils, macrophages, and NK cells. In chickens, CD11c has been found on dendritic cells and macrophages but has not been well studied. DC-pep means peptides that can recognise dendritic cells from a larger leukocyte population. CD40 is a co-stimulatory cell surface receptor from the tumour necrosis factor family. In humans, CD40 is expressed on dendritic cells, monocytes, macrophages, and B cells. In chickens, CD40 has been detected on monocytes, macrophages, DCs, and B cells. CD83 is an immunoglobulin molecule which, in mammals, is used as an activation molecule. In mammals, CD83 has been found on activated NK cells, monocytes, macrophages, neutrophils, dendritic cells, and B cells. In chickens, CD83 has been found on dendritic cells but has not been well studied. DEC-205 is an endocytic receptor which, in mammals, is expressed on DCs, macrophages, and B and T cells. In chickens, DEC-205 has been detected on DCs, B cells, and T cells but has not been well studied. Figure created with BioRender.com.

There is a large choice in methods for targeting antigen-presenting cells which result in the stimulation of different immune responses and therefore affect vaccine-induced protection. Factors affecting the immune response to APC-targeted vaccines include the choice of antigen, receptor, adjuvant, carrier, and administration route. Different combinations of these factors allow for the customisation of vaccines for protection in different species and against different pathogens [107]. Targeting antigens to APCs using a ligand- or antibody-based approach has been shown to increase vaccine efficacy in mammals and birds, with five validated APC targets in birds (Figure 4).

### 5.1. Research into Using Ligand-Based Targeting of APCs

APCs express pattern recognition receptors (PRRs) on their surface and intracellular compartments, which bind to natural ligands commonly expressed on microbes, e.g., the flagellin in bacteria and single-stranded RNA in viruses, also known as pathogen-associated molecular patterns (PAMPs). The conjugation of an antigen to a PRR ligand results in simultaneous antigen processing and stimulation to occur in the same DC [108]. In ligand-based targeting vaccines, different PRRs can be selected to increase the desired immune response. These responses can include the stimulation of different families of T cells or site-specific immune responses in disease-specific organs, e.g., an increase in mucosal immunity [8,108,109].

**Figure 5:**
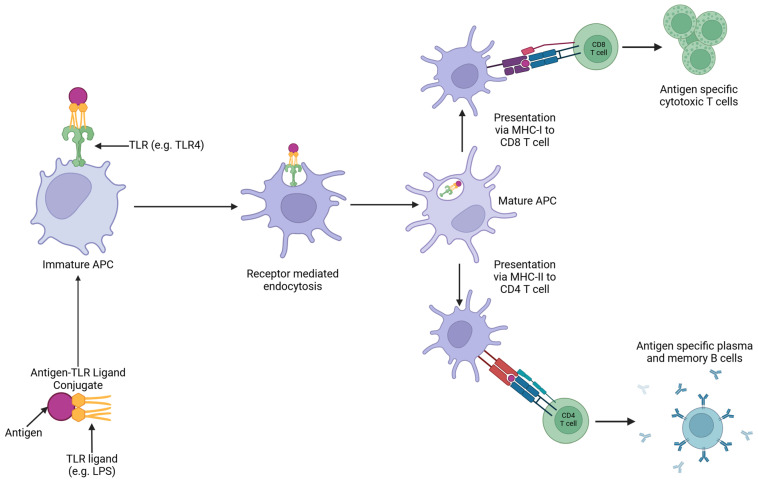
Illustration of ligand-based targeting of APCs. PRR ligands can be chemically or recombinantly bound to antigens. TLR ligands are the most used for antigen-targeting studies as these both enable the targeting of antigens to the APC and have adjuvant properties. The antigen–TLR conjugate vaccine binds to its receptor on the APC; this induces receptor-mediated endocytosis. This induces APC maturation and allows for T-cell presentation via either the MHC-I or MHC-II pathway. MHC-I presentation of the antigen to CD8 T cells results in the development of a population of antigen-specific cytotoxic T cells, which are capable of lysing infected cells. MHC-II presentation of the antigen to CD4 T cells, which present the antigen to B cells, results in the development of antigen-specific antibody production. Created with BioRender.com.

In ligand-based APC-targeting vaccines, the antigen is conjugated to a specific PRR ligand, which results in an antigen–PRR ligand conjugate vaccine. This is then recognised by the associated TLR on an APC and ensures the antigen is processed and then presented via the APC (Figure 5). This increases vaccine potency as it increases antigen presentation and cellular stimulation, resulting in improved associated humoral and cellular immunity [8].

There are four PRR families: TLRs, retinoic acid-inducible gene 1-like helicase receptors (RLRs), oligomerisation domain-like receptors (NLRs), and C-type lectin receptors (CLRs). TLRs are the most studied in antigen–PRR ligand conjugate vaccines in mammals [108]. In poultry, TLR ligands have been studied and used as adjuvants in vaccine development and have shown to be successful. Therefore, these are promising candidates for ligands to use as APC targets (See Section 4.2) [8].

### 5.2. Research into Using Antibody-Based Targeting of APCs in Poultry Vaccines

**Figure 6:**
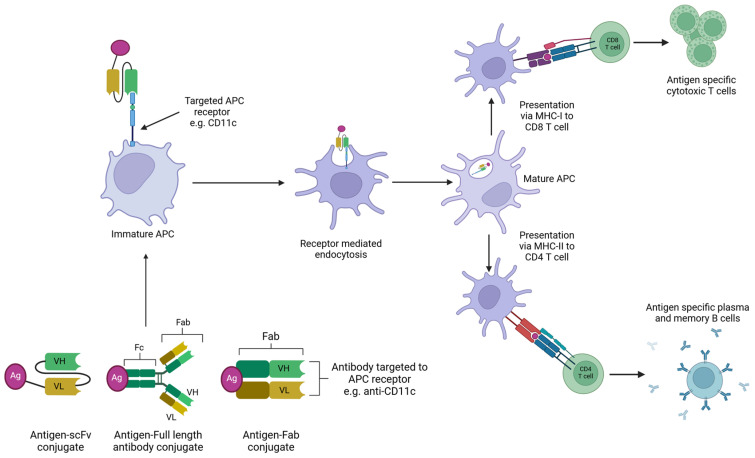
Illustration of ligand-based targeting of APCs. Full antibody or antibody-based fragments can be chemically or recombinantly bound to antigens. APC specificity is determined via the use of anti-receptor antibodies, which target the desired receptor on an APC. The antigen–antibody complex will bind to its targeted receptor on an APC. This induces receptor-mediated endocytosis. This induces APC maturation and allows for T-cell presentation via either the MHC-I or MHC-II pathway. MHC-I presentation of the antigen to CD8 T cells results in the development of a population of antigen-specific cytotoxic T cells, which are capable of lysing infected cells. MHC-II presentation of the antigen to CD4 T cells can activate B cells and results in the development of antigen-specific antibody production. Created with BioRender.com.

Antibody-targeted vaccines use the targeting properties of antibodies to deliver antigens to APCs. This method has been applied to a wide diversity of antigens, as it can accommodate entire proteins or smaller disease-specific peptides. Using antibodies targeted to specific APC receptors decreases the chance of non-specific antigen uptake by irrelevant receptors and thus increases the amount of antigen reaching the APC [8]. Previously, antigens have been chemically conjugated to APC-binding antibodies. More recently, this has been achieved genetically via direct Fab-fragment linkage, including in the tail of the C-terminus of the antibody heavy chain or included in the loops of the constant domain, resulting in APC-specific V-regions on the recombinant antibody-like molecule [20]. Antibody-based targeting of APCs can also be carried out by the conjugation of antigens to specific monoclonal antibodies (mAbs) selected for APC surface molecules or via genetic engineering. In this case, the antigen is fused to antibody fragments, e.g., scFv or fragment antigen-binding regions (Fabs), for specific APC receptors (Figure 6) [8]. Most research into antibody-based targeting of APC vaccines has focused primarily on receptors such as C-type lectin receptors (CLRs), integrins, and Fc receptors. These receptors can also be found on avian APCs. CD11c is present on immature DCs and is a member of the β_2_ integrin family [8]. DEC205 is present on mature DCs and is a CLR [8]. CD83 is a member of the immunoglobulin superfamily and therefore has the potential to act as an Fc receptor, as all known Fc receptors also belong to this family [110,111].

DEC205 and CD11c have been used in antigen–scFv vaccines against AIV in poultry. This method was used to target avian DCs and resulted in faster and higher neutralising antibody production compared with standard non-targeted vaccines. However, CD11c-targeted vaccines also induced increased levels of proinflammatory cytokines, whereas DEC205-targeted vaccines did not [112]. The scFv technology targeting chicken CD83 with the H9 antigen was also found to induce high levels of antibodies in chicks, and vaccination was not affected by the presence of MDAs [113]. This method of vaccination also provided protection when vaccinated animals were challenged in studies with H9N2 virus [114].

In addition, mAb designed to mimic CD40L, which targets CD40, has also been trialled in poultry. CD40 is expressed by chicken macrophages, DCs, B cells, and monocytes. The use of a monoclonal antibody targeting CD40 has also been previously shown to increase antigen-specific IgY responses. When administered orally via the GI tract, significant IgA titres were induced in tracheal mucosa from only one dose of vaccine [115]. Using chicken CD40L has also been studied in poultry vaccination. *Eimeria tenalla* (*E. tenalla*), which causes avian coccidiosis, is usually controlled by drug treatment; however, resistant strains are occurring. *E. tenalla* immune-mapped protein 1 (EtIMP1) was shown to be immunogenic. Therefore, a recombinant EtIMP1–CD40L fusion protein was created and used to immunise chickens, and it induced stronger IgA and IFN-γ responses than the EtIMP1 protein alone [116], demonstrating that the CD40/CD40L family can be used effectively for APC-targeted vaccines in poultry.

An APC-targeted multivalent vaccine has been developed against type 1 and type 2 Bovine Viral Diarrhoea Virus. This vaccine contained a single-chain antibody directed to an MHC class II antigen epitope, which proved effective in challenge studies in reducing clinical signs of disease and decreasing detection of virus in vaccinated calves [117]. Therefore, the ability to develop multivalent vaccines using APC technology in veterinary species is possible, but it has not been trialled in poultry [8,117].

Together, these studies show that APC-targeting vaccines can be administered orally, which may help to stimulate immune responses in disease-specific tissues. However, the choice of receptor that is being targeted needs to be considered, as these can result in different degrees of immune response and therefore may affect their efficacy.

## 6. Research into Using Probiotics in the Enhancement of Vaccines in Poultry

Probiotics are live microorganisms that confer a health benefit through improvements in the intestinal microbial balance. The mode of action of probiotics is not fully understood but has been shown to be beneficial in both humans and farm animals, including chickens [118,119,120].

The gastrointestinal tract (GIT) microbiota plays an important role in the pathogenesis of viruses. Many viral infections occur via mucosal tissues such as the GIT and respiratory system, where microbes encounter commensal microbiota on the surfaces of the tissues. The GIT microbiota is essential for maintaining basal levels of type 1 IFN, which is a critical antiviral response, and it has also been shown that the GIT microbiota helps the host’s immune responses to different pathogens [100,121]. In poultry, the innate immune response to antigens in the GIT is mediated via the caecal tonsils and Peyer’s patches, also referred to as GIT-associated lymphoid tissues (GALTS) [122]. The mechanisms of GIT microbiota-mediated immunity involve PRRs, TLRs, and nod-like receptors (NLRs) [100].

Lactic acid-producing bacteria are already used in the meat and dairy industries. The most common are *Bifidobacterium*, *Lactococcus,* and *Lactobacillus*, some strains of which have probiotic properties. Via mechanisms currently unknown, lactic acid-producing bacteria confer improved immune responses as well as other benefits to the host in several species, including chickens [100,118,120]. The effect of lactic acid-producing bacteria has also been shown to reduce enteric diseases in farms by competitive exclusion mechanisms against pathogenic bacteria and by the production of molecules with bactericidal functions [123]. The importance of the chicken GIT microbiome and its effect on the development of immune responses to viruses is becoming increasingly evident. Interactions between lactobacilli and chicken macrophages have been shown to induce anti-AIV responses including increases in nitric oxide (NO), IFN-γ production, and the expression of the co-stimulatory molecule CD40 [124]. However, probiotic treatment needs to be tailored to the type of vaccination given. It has been shown that *Lactobacillus* spp. enhanced IFN-γ production and increased HAI titres, but only in chickens vaccinated with CpG-adjuvanted whole inactivated H9N2 vaccine, while there was no effect on the immune response in birds vaccinated with non-adjuvanted whole inactivated H9N2 vaccine [125]. This suggests that the mechanisms behind the effects of probiotic treatments still need to be elucidated.

*Bacillus subtilis* (*B. subtilis*) has also been investigated as a mechanism to improve immune responses to AIV vaccination. *B. subtilis* had previously been used as a probiotic treatment in humans and other animal models. The adjuvant effect of *B. subtilis* was investigated on an inactivated H9N2 AIV vaccine in chickens. *B. subtilis* increased the number of B cells and innate cells in the spleen as well as the expression of proinflammatory cytokines such as IL-6 and IL-1β. Restimulation of splenocytes also showed that the adjuvanted vaccine increased the levels of H9N2-specific CD4^+^ and CD8^+^ T-cell proliferation in chickens compared with H9N2 vaccination alone [126].

Vaccination responses against MDV with the HVT vector were also increased with the use of probiotics. The administration of four *Lactobacillus* species on embryonic day 18 and chicks on days 1 to 4 post-hatch increased the expression of MHC-II on spleen macrophages and B cells and decreased spleen T-regulatory (Treg) cells. Levels of IFN-α and IFN-β were also increased in probiotic-treated chicks along with an associated reduction in tumour incidence [127].

Commercially available pre- and/or postbiotic treatments have also been assessed for their effect on the live attenuated *Salmonella Enterititidis* vaccine. In this study, the protection of broiler chickens from challenge with *Salmonella Enterititidis* was investigated after pre- and/or postbiotics were administered via drinking water. This study found that the treatment with pre- or postbiotics at the same time as the live *Salmonella* vaccine reduced mortality rates, vaccine-associated negative growth performance, faecal shedding, and the ability to detect the bacteria in chicken organs such as heart, liver, spleen, and cecum [128].

The method of probiotic administration needs to be considered as there are opposing findings in the literature. One group showed that probiotics given via feed induced higher HI anti-AIV antibodies compared with those given in drinking water [129], whereas a different experiment included probiotics within the vaccination formula itself and showed that this method increased the potency of AIV vaccination [126]. Therefore, the most effective method of probiotic administration to increase vaccine potency still needs to be optimised. There is some evidence of the improvement of vaccine responses in poultry in response to probiotic administration; however, probiotic usage still needs further study.

## 7. Enhancement of Poultry Vaccine Efficacy by the Incorporation of Multiple Antigens

In poultry, there are several multivalent vaccines already in use (Table 1). These include tetravalent vaccines, e.g., avian metapneumovirus, IBV, and NDV (Table 1—VM 08327/3020); quadrivalent vaccines, e.g., avian metapneumovirus, EDS, IBV, and NDV (Table 1—VM 08327/5025); and penta- and octavalent vaccines against different strains of *Eimeria* parasites (Table 1—VM 01708/5101, 01708/4572). Multivalent vaccines are preferred in the commercial poultry industry as they reduce time and labour costs as well as the stress caused by manual handling during the vaccination process itself. However, in multivalent vaccines, potency can be affected by the inclusion of multiple antigens. Interactions between responses to different antigens and the components that make up the multivalent vaccine impair vaccine efficacy. This could lead to inappropriate immune responses due to epitope suppression and/or antigenic competition. The number of antigens and potency therefore needs to be balanced to develop an effective multivalent vaccine [22,130]. This effect has been seen with poultry vaccines, especially when live attenuated vaccines replicate in the same target tissue. Administration of avian metapneumovirus 6 days post-IBV vaccination reduced the efficacy of the avian metapneumovirus [131]. It has also been shown that the combination of more than one HVT-vectored vaccine administered to the same bird resulted in decreased responses to one or both vaccines administered. Here, vaccines towards NDV, ILTV, and IBDV produced in an HVT vector were administered alone or in combination. This study found that the ILTV vaccine was the most affected by the addition of at least one HVT vaccine in combination. This led to increased clinical signs and virus shedding upon challenge. This effect, however, was not significant for the administration of the other HVT-vectored vaccines administered in this study [132].

mRNA technology has allowed for the development of a multivalent nucleoside-modified mRNA lipid nanoparticle vaccine encoding mRNA for 20 independent HA antigens of influenza A (18) and influenza B (2) subtypes. This multivalent vaccine elicited high levels of antibodies that were cross-reactive, providing protection from matched and mismatched influenza challenge in both mice and ferrets [133]. A single H9 HA mRNA vaccine has been trialled in chickens and shown to be safe and effective; therefore, there is the potential that a similar multivalent mRNA AIV vaccine could be trialled and prove effective in poultry [43].

## 8. Conclusions

There is a need to increase the potency of avian vaccines, as vaccination is an essential part of the toolkit to decrease infection and the transmission of disease in poultry. A large amount of research has been carried out investigating methods to increase the immune response to vaccination against several avian diseases; however, progress towards the use of these novel formulations commercially is limited. Further research into the long-term protection elicited by these new technologies also needs to be determined in the field, especially for the vaccination of layers and breeder flocks.

Improvements have been made in traditional adjuvants, such as in adjuvants based on oil-in-water emulsion, which have been shown to increase vaccine potency and are one of the most used adjuvants in commercial poultry vaccines. These are well tolerated and induce increased immune responses compared with their non-adjuvanted counterparts.

However, progress towards the more regular use of novel adjuvants in commercial poultry vaccines is very limited. This is most likely due to the need to optimise and standardise not only the delivery mechanisms but the manufacturing procedures. There is still a worry that novel adjuvants may produce unacceptable side effects in the vaccinated birds. This is especially a concern with the use of immunomodulatory adjuvants such as cytokines and TLRs.

Another issue associated with the wide use of novel technologies is the lack of infrastructure that is needed to scale up the production of new vaccine technologies and adjuvants if the technology is going to be used widely at a low cost. Increased infrastructure for the production of vaccines and adjuvants using novel technologies will be needed in order to maintain a stable vaccine supply and decrease the overall vaccine cost, making it commercially viable for use in poultry.

## Figures and Tables

**Table 1:** List of poultry vaccines licenced in the United Kingdom as per the Veterinary Medicines Directorate, GOV.UK [14] https://www.vmd.defra.gov.uk/productinformationdatabase accessed on 20 November 2024.

Pathogen	Biological Agent	Type of Vaccine	Further Information	Delivery Method	Adjuvant Used	Veterinary Medicine (VM) Number
Avian encephalomyelitis virus	Virus	Live attenuated	Attenuation method unspecified. Strain = 1143 Calnek	Drinking water	None specified	00879/5005
		Live attenuated	Attenuation method unspecified. Strain = 1143 Calnek	Drinking water	None specified	01708/4281
		Live attenuated	Attenuation method unspecified. Strain = AE-6710 Calnek	Drinking water	None specified	42058/5134
		Live (FPV viral vector)	Genetically attenuated. Strain = 1143 Calnek	Wing web	None specified	15052/5029
Avian influenza virus (H5N2)	Virus	Inactivated	Inactivation method unspecified. Strain = A/duck/Potsdam/1402/86	Subcutaneous, intramuscular	Mineral oil for water-in-oil emulsion	01708/5044
Avian metapneumovirus	Virus	Live attenuated	Attenuation method unspecified. Strain = CRR126	Oculonasal, eyedrop, spray	None specified	15052/4086
		Live attenuated	Attenuation method unspecified. Strain = PL21	Water, spray	None specified	08327/5008
		Inactivated	β-propiolactone inactivated. Strain = But 1 #8544	Intramuscular	Mineral oil for water-in-oil emulsion	01708/5105
		Inactivated	Formaldehyde or β-propiolactone inactivated. Strain = But 1 #8544	Intramuscular	Mineral oil for water-in-oil emulsion	01708/5083
		Inactivated	Formaldehyde or β-propiolactone inactivated. Strain = But 1 #8544	Intramuscular	Mineral oil for water-in-oil emulsion	01708/5093
		Live attenuated	Attenuation method unspecified. Strain = 11/94	Oculonasal, spray	None specified	01708/4505
		Inactivated	Inactivation method unspecified. Strain = VCO3	Intramuscular	Mineral oil for water-in-oil emulsion	08327/3023
		Inactivated	Inactivation method unspecified. Strain = VCO3	Intramuscular	Mineral oil for water-in-oil emulsion	08327/5022
		Inactivated	Inactivation method unspecified. Strain = VCO3	Intramuscular	Mineral oil for water-in-oil emulsion	08327/5025
Avian reovirus	Virus	Inactivated	Formalin inactivated. Strain = 1733 and 2408	Intramuscular, subcutaneous	Mineral oil for water-in-oil emulsion	01708/4329
Chicken anaemia virus	Virus	Live	Apathogenic strain (for adult birds). Strain = Cux-1	Drinking water	None specified	00879/5042
		Live attenuated	Attenuation method unspecified. Strain = 26P4	Intramuscular, subcutaneous	dl-α-tocopherol acetate	01708/4322
Coccidia (*Eimeria*)	Parasite	Live attenuated	Attenuation method unspecified. Strain = 044, 013, 007, 004	In ovo	None specified	17533/5014
		Live	Precocious strain (for adult birds). Strain = RA-3+20, MCK+10, Jormit 3+9, Rt 3+15	Spray, feed, drinking water	None specified	30282/5018
		Live	Precocious strain (for adult birds). Strain = mednec 3+8, roy 3+28	Spray, feed, drinking water	None specified	30282/4032
		Live	Precocious strain (for adult birds). Strain = HP, CP, MFP	Spray, feed, drinking water	None specified	01708/5101
		Live	Precocious strain (for adult birds). Strain = HP, CP, MFP	Spray, feed, drinking water	None specified	01708/4572
*E. coli*	Bacteria	Live attenuated	Genetically modified. Strain = O78-EC34195	Spray, drinking water	None specified	42058/5046
		Inactivated	Inactivation method unspecified. F11-antigen (fimbrial antigen), FT-antigen (flagellar toxin antigen)	Intramuscular, subcutaneous	Mineral oil for water-in-oil emulsion	01708/5085
Egg drop syndrome (EDS)	Virus	Inactivated	Inactivation method unspecified. Strain = V127	Intramuscular	Mineral oil for water-in-oil emulsion	08327/5012
		Inactivated	Inactivation method unspecified. Strain = V127	Intramuscular	Mineral oil for water-in-oil emulsion	08327/5025
		Inactivated	Inactivation method unspecified. Strain = BC14	Intramuscular, subcutaneous	Mineral oil for water-in-oil emulsion	01708/4275
		Inactivated	β-propiolactone inactivated. Strain = BC14	Intramuscular	Mineral oil for water-in-oil emulsion	01708/5083
Fowlpox virus (FPV)	Virus	Live viral vector	Genetically attenuated. Strain = rFP-LT, expressing fusion protein gene and encapsidation protein gene of ILT	Wing web	None specified	15052/5029
		Live viral vector	Genetically attenuated. Strain = rFP-LT, expressing fusion protein gene and encapsidation protein gene of ILT	Wing web	None specified	15052/5028
Infectious bronchitis virus (IBV)	Virus	Live attenuated	Attenuation method unspecified. Strain = V-173/11	Spray, oculonasal, drinking water	None specified	43676/4005
		Live attenuated	Attenuation method unspecified. Strain = H-20	Spray, oculonasal, drinking water	None specified	43676/4002
		Live attenuated	Attenuation method unspecified. Strain = 1/96	Spray, oculonasal, drinking water	None specified	15052/5043
		Live attenuated	Attenuation method unspecified. Strain = B-48	Spray	None specified	15052/4088
		Inactivated	Inactivation method unspecified. Strain = Mass41	Intramuscular	Mineral oil for water-in-oil emulsion	08327/5012
		Inactivated	Inactivation method unspecified. Strain = Mass41	Intramuscular	Mineral oil for water-in-oil emulsion	08327/5025
		Live attenuated	Attenuation Method Unspecified. Strain = CR88121	Spray	None specified	08327/4265
		Live attenuated	Attenuation Method Unspecified. Strain = H120	Spray	None specified	08327/5005
		Inactivated	Inactivation method unspecified. Strain = M41	Intramuscular, subcutaneous	Mineral oil for water-in-oil emulsion	01708/4275
		Live attenuated	Attenuation method unspecified. Strain = 4–91	Spray, intraocular/nasal, drinking water	None specified	01708/5043
		Live attenuated	Attenuation method unspecified. Strain = Ma5	Spray, intraocular/nasal, drinking water	None specified	01708/4283
		Live attenuated	Attenuation method unspecified. Strain = D388	Spray, intraocular/nasal	None specified	01708/5042
		Live attenuated	Attenuation method unspecified. Strain = Ma5	Spray, intraocular/nasal, drinking water	None specified	01708/4315
		Inactivated	Formaldehyde or β-propiolactone inactivated. Strain = M41, 249 g	Intramuscular	Mineral oil for water-in-oil emulsion	01708/5093
		Inactivated	Formaldehyde or β-propiolactone inactivated. Strain = M41, 249 g	Intramuscular	Mineral oil for water-in-oil emulsion	01708/5083
		Live attenuated	Attenuation method Unspecified. Strain = H120	Spray, intraocular/nasal, drinking water	None specified	42058/5121
		Live attenuated	Attenuation method unspecified. Strain = H120, D274	Spray, drinking water	None specified	42058/4103
		Live attenuated	Attenuation method unspecified. Strain = L1148	Spray	None specified	42058/5103
		Live attenuated	Attenuation method unspecified. Strain = Ma1263, Ak3168	Spray	None specified	42058/5140
Infectious bursal disease (IBD)	Virus	Live attenuated	Attenuation method unspecified. Strain = LC75	Drinking water	None specified	00879/4187
		Live attenuated	Attenuation method unspecified. Strain = G6	Drinking water	None specified	43676/5000
		Live attenuated	Attenuation method unspecified. Strain = G61, Winterfield 2512	Drinking water	None specified	15052/4059
		Live attenuated	Attenuation method unspecified. Strain = Winterfield 2512	In ovo, subcutaneous	None specified	15052/4030
		Live attenuated	Attenuation method unspecified. Strain = S706	Spray, drinking water	None specified	08327/4192
		Live attenuated	Attenuation method unspecified. Strain = S706	Drinking water	None specified	08327/4323
		Live attenuated	Attenuation method unspecified. Strain = 1052	In ovo, subcutaneous	None specified	17533/5005
		Live attenuated	Attenuation method unspecified. Strain = CH/80	Drinking water	None specified	17533/5017
		Live attenuated	Attenuation method unspecified. Strain = GM97	Drinking water	None specified	17533/4002
		Live (HVT viral vector)	Genetic recombination, HVT vector for MDV, IBDV, and ILT vaccination	In ovo, subcutaneous	None specified	01708/5082
		Live (HVT viral vector)	Genetic recombination, HVT vector for MDV, NDV and IBDV vaccination	In ovo, subcutaneous	None specified	01708/5040
		Live attenuated	Attenuation method unspecified. Strain = G61, Winterfield 2512	In ovo, subcutaneous	None specified	15052/4154
		Live attenuated	Attenuation method unspecified. Strain = 228E	Drinking water	None specified	01708/4333
		Live attenuated	Attenuation method unspecified. Strain = D78	Spray, intraocular/nasal, drinking water	None specified	01708/4237
		Inactivated	Formaldehyde or β-propiolactone inactivated. Strain = D78	Intramuscular	Mineral oil for water-in-oil emulsion	01708/5093
		Live attenuated	Attenuation method unspecified. Strain = SYZA26	Subcutaneous	None specified	15052/5032
		Live attenuated	Attenuation method unspecified. Strain = V877	Drinking water	None specified	42058/5138
		Live attenuated	Attenuation method unspecified. Strain = Lukert	Spray, drinking water	None specified	42058/5137
		Live (HVT viral vector)	Genetic recombination, HVT vector for MDV and IBDV	In ovo, subcutaneous	None specified	04491/5043
		Live (HVT viral vector)	Genetic recombination, HVT vector for MDV, NDV, and IBDV	In ovo, subcutaneous	None specified	15052/5001
		Live (HVT viral vector)	Genetic recombination-HVT vector for MDV and IBDV	In ovo, subcutaneous	None specified	04491/5060
Infectious laryngotracheitis (ILT)	Virus	Live (HVT viral vector)	Genetic recombination, HVT vector, gD and gl glycoproteins. Strain = 138	Subcutaneous	None specified	01708/5039
		Live (HVT viral vector)	Genetic recombination, HVT vector for MDV, IBDV, and ILT vaccination	In ovo, subcutaneous	None specified	01708/5082
		Live (HVT viral vector)	Genetic recombination, HVT vector for MDV, NDV, and ILT vaccination	In ovo, subcutaneous	None specified	01708/5041
		Live attenuated	Attenuation method unspecified. Strain = Salsbury 146	Intraocular	None specified	42058/4106
		Live (FPV viral vector)	Genetically attenuated. Strain = rFP-LT, expressing fusion protein gene and encapsidation protein gene of ILT	Wing web	None specified	15052/5029
		Live (FPV viral vector)	Genetically attenuated. Strain = rFP-LT, expressing fusion protein gene and encapsidation protein gene of ILT	Wing web	None specified	15052/5028
Marek’s disease virus (MDV)	Virus	Live (HVT viral vector)	Genetic recombination-HVT vector for MDV vaccination. Strain = FC 126	In ovo, subcutaneous	None specified	15052/4089
		Live attenuated	Attenuation method unspecified. Strain = CVI-988	Subcutaneous	None specified	15052/4151
		Live attenuated	Attenuation method unspecified. Strain = CVI-988	Subcutaneous, intramuscular	None specified	01708/4294
		Live (HVT viral vector)	Genetic recombination, HVT vector for MDV vaccination. Strain = FC 126, CVI-988	In ovo, subcutaneous	None specified	01708/4354
		Live attenuated	Attenuation method unspecified. Strain = CVI-988	Intramuscular	None specified	42058/4107
		Live (HVT viral vector)	Genetic recombination, HVT vector for MDV vaccination. Strain = FC 126, CVI-988	Subcutaneous, intramuscular	None specified	42058/4108
		Live attenuated	Attenuation method unspecified. Strain = RN1250	Subcutaneous	None specified	04491/5042
		Live (HVT viral vector)	Genetic recombination, HVT vector for MDV and IBDV	In ovo, subcutaneous	None specified	04491/5043
		Live (HVT viral vector)	Genetic recombination, HVT vector for MDV, IBDV, and ILT vaccination.	In ovo, subcutaneous	None specified	01708/5082
		Live (HVT viral vector)	Genetic recombination, HVT vector for MDV, NDV, and IBDV vaccination.	In ovo, subcutaneous	None specified	01708/5040
		Live (HVT viral vector)	Genetic recombination, HVT vector for MDV, NDV, and ILT vaccination.	In ovo, subcutaneous	None specified	01708/5041
		Live (HVT viral vector)	Genetic recombination, HVT vector for MDV, NDV, and IBDV.	In ovo, subcutaneous	None specified	15052/5001
		Live (HVT viral vector)	Genetic recombination, HVT vector for MDV and IBDV.	In ovo, subcutaneous	None specified	04491/5060
		Live	Apathogenic strain (for adult birds). Strain = FC126	Intramuscular, subcutaneous	None specified	01708/4289
*Mycoplasma gallisepticum*; *Mycoplasma synovia*	Bacteria	Inactivated	Inactivation method unspecified. Strain = MS-NEV1, MS-NEV2	Subcutaneous	Mineral oil for water-in-oil emulsion	43877/5001
		Inactivated	Inactivation method unspecified. Strain = MG-NEV40, MG-NEV45	Subcutaneous	Mineral oil for water-in-oil emulsion	43877/5000
		Live attenuated	Attenuation method unspecified. Strain = MS-H	Intraocular	None specified	42983/5000
		Live attenuated	Attenuation method unspecified. Strain = MG 6/85	Spray	None specified	01708/5103
		Live attenuated	Attenuation method unspecified. Strain = MS1	Spray	None specified	01708/4607
		Live attenuated	Attenuation method unspecified. Strain = VG/GA-AVINEW 5.5	Intraocular	None specified	08327/5043
Newcastle disease (NDV)	Virus	Live attenuated	Attenuation method unspecified. Strain = 13–1	Intraocular, spray, drinking water	None specified	00879/5035
		Live lentogenic	Strain = Hitchner B1	Intraocular/nasal, spray, drinking water	None specified	43676/4003
		Live lentogenic	Strain = La Sota	Intraocular/nasal, spray, drinking water	None specified	43676/4000
		Inactivated	Inactivation method unspecified. Strain = Ulster 2C	Intramuscular	Mineral oil for water-in-oil emulsion	08327/5012
		Inactivated	Inactivation method unspecified. Strain = Ulster 2C	Intramuscular	Mineral oil for water-in-oil emulsion	08327/5025
		Live (HVT Viral Vector)	Genetic recombination, HVT vector for MDV, NDV, and IBDV vaccination.	In ovo, subcutaneous	None specified	01708/5040
		Live (HVT Viral Vector)	Genetic recombination, HVT vector for MDV, NDV, and ILT vaccination.	In ovo, subcutaneous	None specified	01708/5041
		Inactivated	Inactivation method unspecified. Strain = Clone 30	Intramuscular, subcutaneous	Mineral oil for water-in-oil emulsion	01708/4275
		Live attenuated	Attenuation method unspecified. Strain = Clone 30	Spray, intraocular/nasal, drinking water	None specified	01708/4315
		Live attenuated	Attenuation method unspecified. Strain = C2	Spray, intraocular/nasal	None specified	01708/5088
		Live attenuated	Attenuation method unspecified. Strain = Clone 30	Spray, intraocular/nasal, drinking water	None specified	01708/4276
		Inactivated	Formaldehyde or β-propiolactone inactivated. Strain = Clone 30	Intramuscular	Mineral oil for water-in-oil emulsion	01708/5093
		Inactivated	Formaldehyde or β-propiolactone inactivated. Strain = Clone 30	Intramuscular	Mineral oil for water-in-oil emulsion	01708/5083
		Live attenuated	Attenuation method unspecified. Strain = sm24/Rif12/Ssq, Nal2/Rif9/Rtt	Drinking water	None specified	00879/4188
*Salmonella* (enteritidis, typhimurium, and infantis)	Bacteria	Live attenuated	Attenuation method unspecified. Strain = sm24/Rif2/Ssq,	Drinking water	None specified	00879/4189
		Live attenuated	Attenuation method unspecified. Strain = Nal2/Rif9/Rtt	Drinking water	None specified	00879/5037
		Live attenuated	Genetically attenuated. Strain = 441/014	Drinking water	None specified	15052/5045
		Inactivated	Inactivation method unspecified. Strain = PT4, DT104, S03499-06	Intramuscular	Aluminium hydroxide	01708/5073
		Inactivated	Inactivation method unspecified. Strain = PT4, DT104	Intramuscular	Aluminium hydroxide	01708/5114
		Live attenuated	Attenuation method unspecified. Strain = ST CAL 10 Sm+/Rif+/Ssq-	Drinking water	None specified	20634/5002
		Live attenuated	Attenuation method unspecified. Strain = ST CAL 16 Str+/Rif+/Enr-	Drinking water	None specified	20634/5000
